# COL3A1 and Its Related Molecules as Potential Biomarkers in the Development of Human Ewing's Sarcoma

**DOI:** 10.1155/2021/7453500

**Published:** 2021-12-22

**Authors:** Min Tang, Peiqing Liu, Xiaoke Wu, Jie Gong, Jiacheng Weng, Guangyu Gao, Yulong Liu, Lei Gan

**Affiliations:** ^1^Department of Radiotherapy and Oncology, First People's Hospital of Kunshan, Kunshan, Jiangsu Province, China; ^2^Department of Oncology, The Second Affiliated Hospital of Soochow University, Suzhou 215004, China; ^3^State Key Laboratory of Radiation Medicine and Protection, School of Radiation Medicine and Protection, Soochow University, Suzhou 215123, China; ^4^Collaborative Innovation Center of Radiological Medicine of Jiangsu Higher Education Institutions, Suzhou 215123, China

## Abstract

**Background:**

Ewing's sarcoma (ES) is the most common malignant primary bone tumor in children and adolescents. This study is aimed at developing new prognostic markers and building a microRNA-mRNA network in the development of ES.

**Method:**

GSE80201 and GSE39262 were downloaded from the Gene Expression Omnibus (GEO) database. Bioinformatics analysis was used to download and process data. The coexpression of differentially expressed microRNAs (DEMs) and genes (DEGs) was selected by using R software. The FunRich database was utilized to perform cellular component (CC), molecular function (MF), and biological process (BP) enrichment analysis. Cytoscape and ClueGO were used to perform Kyoto Encyclopedia of Genes and Genomes (KEGG) enrichment analysis and construct the mRNA-microRNA network. The Kaplan-Meier Plotter was used to perform prognosis analysis between the expression level of genes we selected and overall survival (OS) of patients with ES. Univariate analysis and multivariate analysis were carried out to research the prognostic value of identified mRNA expression in ES according to TCGA database.

**Results:**

By using bioinformatics analysis, 10 DEMs and 5 target mRNAs were identified. Based on the KmPlot software, COL1A2, COL3A1, and TGFBI were significantly related to the OS of patients with ES. High COL3A1 mRNA expression was correlated with distant metastasis, margin status, and poor overall survival of ES. Besides, multivariate analysis indicated that COL3A1 was an independent risk factor for ES patients.

**Conclusions:**

In conclusion, our results suggest that COL3A1 and its related molecules may be a potential diagnostic and prognostic biomarker for patients with ES.

## 1. Background

Ewing's sarcoma (ES) is thought to originate from mesenchymal stem cells in pediatric patients [1]. It is the second most common osteosarcoma in pediatric patients and young people [2]. ES can appear as conventional ES or extraosseous ES and is now classified as undifferentiated small round cell sarcoma of bone and soft tissue [3]. The incidence age of the disease is 10~15 years old. Boys are more common than girls. This kind of tumor has high malignancy, easy recurrence, and poor prognosis. Bone ES often occurs in the ilium, femur, humerus, fibula, and tibia. When invading long tubular bone, it mostly occurs in the shaft. Compared with bone ES, extraosseous Ewing sarcoma (EES) occurred mainly in the trunk and central axis [4]. At present, the treatment methods of ES are multidisciplinary comprehensive treatment strategies such as chemotherapy, surgery, and radiotherapy. The multidisciplinary team should also include nursing, nutrition, psychology, and rehabilitation disciplines [5]. With the in-depth study of the occurrence, development, and metastasis mechanism of Ewing sarcoma, the molecular targeted therapy of Ewing sarcoma has gradually become a new research hotspot. At present, it mainly focuses on targeted silencing of EWS/FLI-1 fusion gene, targeted blocking of insulin-like factor receptor, targeted inhibition of receptor tyrosine kinase, antiangiogenesis, and so on [6]. The study of new therapeutic targets is of great significance for the treatment of Ewing sarcoma.

miRNA is a kind of noncoding single-stranded RNA with a length of 18~25 nucleotides. It not only plays a very important role in gene expression regulation but also plays an important role in many biological processes such as cell proliferation, differentiation, apoptosis, and hematopoiesis [7]. A large number of studies have shown that the expression level of miRNA is closely related to the occurrence and development of many kinds of tumors. The monitoring of miRNA expression level is of great significance in the early diagnosis and prognosis of diseases [8]. In particular, Zhai et al. found that microRNA-181 is a potential molecular biomarker in the clinical management of glioblastoma and is associated with poor prognosis in patients [9]. Zhou et al. reported that microRNA-134-mediated direct downregulation of laminin subunit gamma 2 inhibits migration and invasion of cancer stem cells in oral squamous cell carcinoma by suppressing the PI3K-Akt signaling pathway [10]. Jiang et al. also reported that miR-24 was significantly suppressed in gastric cancer tissues; it can inhibit the proliferation, migration, and invasion and enhances chemosensitivity of human gastric carcinoma by targeting DND microRNA-mediated repression inhibitor 1. Besides, it may be an important therapeutic target for the treatment of gastric carcinoma [10].

In this article, microarray data from the GEO database and ES clinical sample information in TCGA database were used for identifying differently expressed microRNAs (DEMs) between normal tissues and ES tissues. By using a variety of mRNA and microRNA-related functional databases and performing verification experiments, several genes associated with the development of ES and subsequent pathways were identified.

## 2. Methods

### 2.1. Microarray Data

The GEO database, with the full name Gene Expression Omnibus, is a gene expression database created and maintained by the National Biotechnology Information Center (NCBI). It was founded in 2000 and contains high-throughput gene expression data submitted by research institutions around the world. In our article, gene expression profile data (GSE80201 and GSE39262) were obtained from GEO.

GSE80201 had biopsies from 20 ES patients using paraffin-embedded tissues and normal human mesenchymal stromal cells as controls. MicroRNA expression profiling analysis of these samples was performed on miRXplore TM Microarray (968) (GPL17603). Dataset GSE149507 includes 46 sarcoma cell lines and 5 primary cell lines. Gene expression profiling was performed on Affymetrix Human Genome U133A Array (GPL96).

### 2.2. Differentially Expressed miRNA Analysis

GEO2R, an R-associated web application, was applied to filter DEMs between normal tissues and ES tissues. We also used R software to analyze two sets of data. *p* < 0.05 and ∣logFC | ⩾2 were considered as cutoff criterion.

### 2.3. Gene Ontology and Pathway Enrichment Analysis

The FunRich database is an online database. Users can upload miRNA and mRNA online for target gene prediction, gene enrichment analysis, and pathway correlation analysis. At the same time, they can also convert ID online and present the results in various forms of pictures. In addition to the annotation of the function of genes, we also know that genes will participate in various pathways of the human body. The database based on human pathways is the pathway-related database. KEGG is a kind of path-related database. Cytoscape software and ClueGO were used to perform this study and build a microRNA-mRNA network. ClueGO: deciphering and enriching go and pathways can visually summarize similar processes or pathways, mainly GO and KEGG, and the author can set his threshold to dynamically change the network. ClueGO has two main features: (1) according to the gene list, it can be used for the visualization of terms and (2) the comparison of the functional interpretation of the two clusters. Based on the above software, the results of data processing are presented in the form of a network diagram.

### 2.4. Prediction of miRNA Target Genes and miRNA-mRNA Regulatory Network

miRNA is differentially expressed in a variety of tumors compared with normal tissues. Its mechanisms include miRNA is located in the cancer-related genomic region (CAGR), epigenetic regulation of miRNA expression, and developmental abnormalities of miRNA processing genes and proteins. These mechanisms enable miRNA not only to induce cancer but also to inhibit tumorigenesis. Differentially expressed microRNAs were uploaded to the FunRich software to obtain target mRNAs. Besides, GSE39262 was analyzed by using R software. According to the prediction results of target genes in FunRich software and the DEGs of GSE39262, the coexpressed genes between the two results were identified, and the regulatory network was built by using Cytoscape.

### 2.5. Analysis of the Relationship between the Expression Level of mRNAs and Prognosis of Patients with ES

KmPlot is an online tool for survival analysis. It was originally designed to analyze the survival of miRNA in liver cancer. It can collect miRNA expression profile data related to liver cancer from large databases such as TCGA and GEO, sort out the reported survival-related biomarker miRNAs from the literature for survival analysis, and further screen biomarker miRNAs by integrating the results of Cox regression and differential expression. Later, it was further expanded on this basis. At present, it supports the survival analysis of 21 tumor types, including miRNA and mRNA. In this study, patients with ES were divided into two groups. By inputting the mRNAs we screened into the website, we can get the corresponding survival curve.

### 2.6. Gene Expression and Clinical Characteristics in TCGA

The associated statistics offered by TCGA are open and do not need the approval of the local ethics committee. The data of 1145 patients with small cell lung cancer were obtained from TCGA database. COL1A2, COL3A1, and TGFBI mRNA level, clinicopathological data, and general data of patients with ES were collected.

## 3. Results

### 3.1. Identification of the DEGs and DEMs between ES Samples and Normal Samples

**Figure 1:**
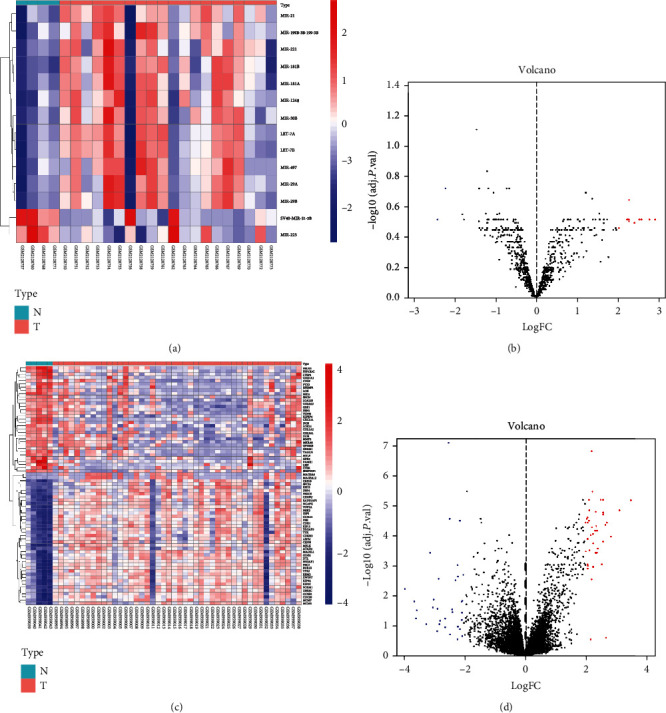
Heat map and volcano map of differentially expressed genes of GSE80201 and GSE39262. (a) Heat map of DEGs in GSE80201. (b) Volcano map of DEGs in GSE80201. (c) Heat map of DEGs in GSE39262. (d) Volcano map of DEGs in GSE39262. Red dots represent upregulated genes, and blue dots represent downregulated genes.

GEO2R was utilized to analyze the microRNA and target gene expression profiles from the GSE80201 and GSE39262. According to the cutoff criteria (*p* < 0.05 and ∣log_2_FC | ≥2), 82 DEMs including miRNA-181b, miRNA-29A, miRNA-223, miRNA-21, miRNA-29B, miRNA-181A, miRNA-30B, and miRNA-1248 and 74 DEGs were identified (Figure 1).

### 3.2. Gene Ontology Enrichment Analysis

**Figure 2:**
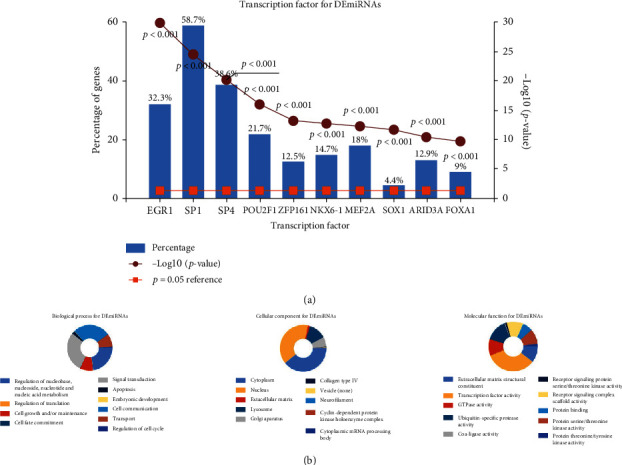
Gene Ontology enrichment. (a) Identification of the potential transcription factors of DEMs by FunRich software. (b) The top 10 of biological process, cellular component, and molecular function of the target genes of miRNAs.

**Figure 3:**
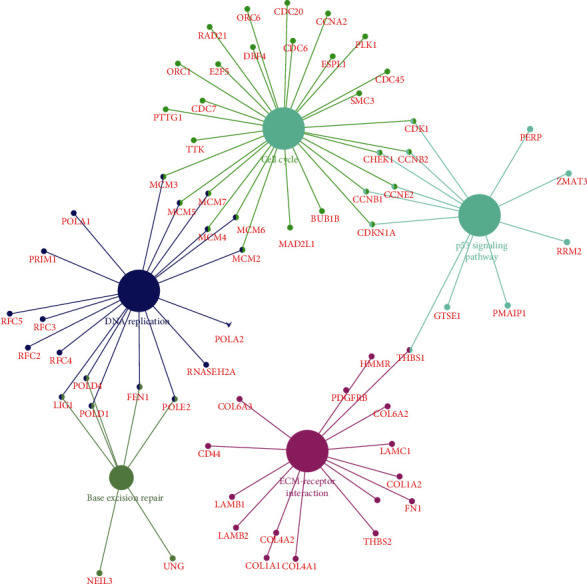
KEGG pathway enrichment analysis of potential target mRNAs.

Transcription factor enrichment analysis was conducted by using FunRich software, and the result is shown in Figure 2(a). To further learn about the mechanisms of identified microRNAs, it was also used to perform Gene Ontology enrichment analysis. The pictures demonstrated that DE-microRNAs were most enriched in the regulation of nucleobase, nucleoside, regulation of translation, extracellular matrix, Golgi apparatus, cyclin-dependent protein kinase holoenzyme complex, extracellular matrix structural constituent, transcription factor activity, and GTPase activity (Figure 2(b)). Furthermore, Cytoscape and ClueGO were used to conduct KEGG pathway enrichment analysis. These selected microRNAs were mainly enriched in 8 pathways: p53 signaling pathway, ECM-receptor interaction, autophagy, DNA replication, base excision repair, complement and coagulation cascades, homologous recombination, and nucleotide excision repair (Figure 3).

### 3.3. microRNA-mRNA Regulatory Network

**Figure 4:**
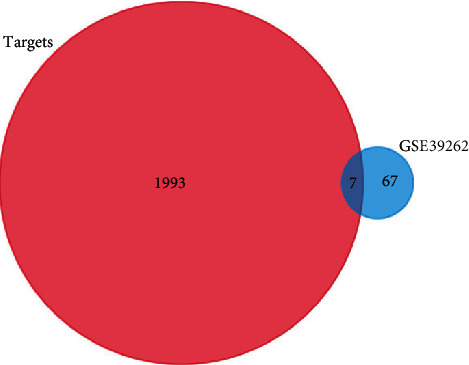
Venn diagram of GSE80201 and GSE39262.

**Figure 5:**
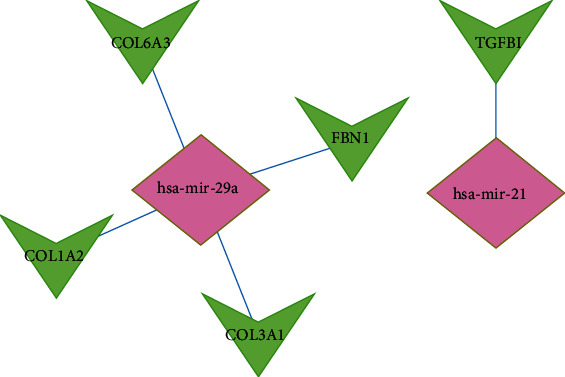
Identified target mRNAs and miRNA-mRNA regulatory network.

Based on FunRich software, 2000 target genes were downloaded and 7 of them were differentially expressed in GSE39262 (DBF4, CEP55, FBN1, COL1A2, COL3A1, TGFBI, and COL6A3) (Figure 4). According to the relationship between them, 5 essential miRNA-mRNA pairs (microRNA-29a, microRNA-21, FBN1, COL1A2, COL3A1, TGFBI, and COL6A3) were selected which were identified for further study (Figure 5).

### 3.4. Analysis of the Gene Expression and Their Relationships with ES Prognosis

**Figure 6:**
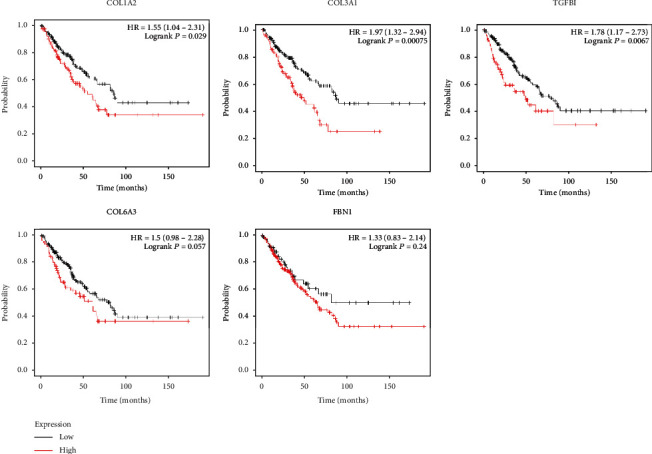
The association between the expression level of selected target mRNAs and Ewing sarcoma prognosis.

KmPlot was utilized to research the survival of patients with ES. By submitting the 5 genes we selected, survival curves were obtained. The results indicated that COL1A2, COL3A1, and TGFBI (Figure 6) were significantly related to the prognosis of patients with ES. However, the expression level of FBN1 and COL6A3 may have no significant association with OS.

### 3.5. Correlation between Clinical Characteristics and COL1A2, COL3A1, and TGFBI mRNA Expression of ES

Clinical and gene expression data of 101 ES were obtained from TCGA database, including metastasis stage, tumor region, age stage, gender stage, and race stage. We found that the expression level of TGFB1 was not associated with metastasis stage, tumor region, age stage, gender stage, and race stage (*p* > 0.01) (Table 1). Besides, relationships between clinical characteristics and TGFB1 expression level in ES were researched. The univariate analysis revealed that the metastasis stage was related to overall survival. However, higher TGFB1 mRNA expression, tumor region, age stage, gender stage, and primary site progression were not related to overall survival. Multivariate analysis also indicated that only the metastasis stage was an independent risk factor for OS in ES (Table 2). Our results also demonstrated that the expression level of COL1A2 was not associated with metastasis stage, tumor region, age stage, gender stage, and race stage (*p* > 0.01) (Table 3). Besides, correlations between clinical characteristics and COL1A2 expression in ES were researched. The univariate analysis revealed that the metastasis stage was related to overall survival. However, higher COL1A2 mRNA expression, tumor region, age stage, gender stage, and primary site progression were not correlated with overall survival. Multivariate analysis also showed that only the metastasis stage was an independent risk factor for overall survival in ES (Table 4). We also found that the expression level of COL3A1 was not associated with metastasis stage, tumor region, age stage, gender stage, and race stage (*p* > 0.01) (Table 5). Besides, correlations between clinical characteristics and COL3A1 expression in ES were researched. The univariate analysis revealed that metastasis stage and COL3A1 expression level are related to overall survival. However, tumor region, age stage, gender stage, and primary site progression were not related to overall survival. Multivariate analysis also indicated that COL3A1 expression level and metastasis stage were an independent risk factor for OS in ES (Table 6).

## 4. Discussion

Up to the present, cancer has become the first-rate killer in the world. Despite tremendous efforts being made to ameliorate tumor treatment, cancer cases are increasing every year [11, 12]. In our article, GSE80201 and GSE39262 were downloaded from the GEO database. 82 DEMs including microRNA-181b, microRNA-29A, microRNA-223, microRNA-21, microRNA-29B, microRNA-181A, microRNA-30B, and microRNA-1248 and 74 DEGs were identified. To further understand the mechanisms of the 5 microRNAs in ES, we used FunRich for the next research. GO and KEGG analysis showed that these microRNAs were primarily related to the regulation of nucleobase, nucleoside, regulation of translation, extracellular matrix, Golgi apparatus, cyclin-dependent protein kinase holoenzyme complex, extracellular matrix structural constituent, transcription factor activity, and GTPase activity. This is consistent with the recognition that lysosomes and nucleus play a key role in several human diseases, such as cancer, obesity, neurodegenerative diseases, and infection [13]. As for transporter activity, it is involved in various tumor metastases, targeting lactate transporters and drugs to treat cancer and may serve as an opportunity to develop new therapies for inflammation and cancer [14, 15]. Besides, KEGG research indicated that these genes were mainly enriched in 8 pathways including p53 signaling pathway, ECM-receptor interaction, autophagy, DNA replication, base excision repair, complement and coagulation cascades, homologous recombination, and nucleotide excision repair, which were shown to affect migration and proliferation [16]. In many human tumors, p53 function is destroyed by p53 gene mutation and other mechanisms, including the amplification and/or overexpression of p53 negative regulators (such as murine double minute 2 and murine double minute 4), which is a prerequisite for the occurrence and/or progression of many human tumors [17, 18]. To ensure the proper function of p53 in regulating many basic cellular processes, p53 protein level and activity are strictly regulated in cells. Under nonstress conditions, p53 protein in normal cells is usually maintained at a low level, but the half-life of p53 protein increases significantly, resulting in various stress signals (such as DNA damage, hypoxia, nutritional deficiency, and oncogene activation) inside and outside cells. Once activated, p53 binds to the p53 response element in the target gene and regulates its expression in a transcriptional manner [19]. The expression of endogenous p53 is silent in different kinds of tumors. Polyphenols from various dietary sources, including luteolin, quercetin, and epigallocatechin-3 gallate, can increase the expression of p53 in several tumor cell lines through different mechanisms. Polyphenols can stabilize p53 protein by p53 phosphorylation, p53 acetylation, and reducing oxidative stress. Previous articles also linked p53 mutation with chemotherapy resistance, and polyphenols overcome the chemotherapy resistance of tumor cells by increasing the expression of p53 [20, 21]. As for extracellular matrix-receptor interaction, it was also the most differentially expressed gene-enriched signaling pathway. Its pathway-related genes play a key role in the process of tumor abscission, adhesion, degradation, movement, and proliferation. The role of the extracellular matrix in other tumors has been demonstrated. The extracellular matrix is upregulated in prostate carcinoma [22] and takes part in the development of cancer invasion and metastasis in gastric carcinoma [23]. Besides, the colorectal cancer extracellular matrix can promote the occurrence of epithelial-mesenchymal transformation (EMT) [24]. Glioblastoma is the most common adult brain cancer. The pathological features were abnormal neovascularization and diffuse infiltration of cancer cells. The relationship between ECM and glioblastoma microenvironment is very vital in this development [25]. By utilizing FunRich software, 2000 potential target mRNAs were downloaded and 7 of them were differentially expressed in GSE39262 (DBF4, CEP55, FBN1, COL1A2, COL3A1, TGFBI, and COL6A3). According to the relationship between them, 5 essential miRNA-mRNA pairs (microRNA-29a, microRNA-21, FBN1, COL1A2, COL3A1, TGFBI, and COL6A3) were selected.

The human miRNA-29 family has 3 members including microRNA-29a, microRNA-29b, and microRNA-29c. Among the members of the microRNA-29 family, microRNA-29a was firstly found by Rauhut [26]. Researches about microRNA expression in cancer tissues or cell lines showed that microRNA-29 was downregulated in most carcinomas and upregulated in a few carcinomas. The abnormal expression of microRNA-29 and the carcinogenic or antitumor function of microRNA-29 have been widely studied in many kinds of carcinomas [27–30]. A previous study reported that the microRNA-29 family (microRNA-29a, microRNA-29b, and microRNA-29c) inhibited several proteins related to invasion and metastasis of lung carcinoma. In the nervous system, microRNA-29 was discovered to be downregulated in nervous system tumors such as glioblastoma and neuroblastoma [31, 32]. Besides, in the musculoskeletal system, microRNA-29 was discovered downregulated in osteoblast tumors. microRNA-29a induces osteoblast apoptosis by silencing B cell lymphoma-2 and myeloid cell leukemia 1 and inducing E2F transcription factor 1 and E2F transcription factor 3 expression [33].

miRNA-21 is a member of the miRNA family and encoded by the MIR21 gene on human chromosome 17q23.2. Mature microRNA-21 is formed from endogenous noncoding RNA molecules of about 22 nucleotides and integrated into RNA-induced silencing complex, which binds to 3′-untranslated regions of different genes by incomplete base pairing with microRNA. The expression level of microRNA-21 is overexpressed in plenty of solid tumors, including lung carcinoma, colorectal carcinoma, and gastric carcinoma [34–36]. In addition, previous researches have shown that microRNA-21 is also overexpressed in immune cells, promoting immune-related inflammatory diseases and taking part in the pathogenesis of autoimmune diseases [37].

KmPlot was utilized to study the OS of patients with ES. By submitting the 5 genes we selected, 5 survival curves were obtained. The results showed that COL1A2, COL3A1, and TGFBI were significantly related to the prognosis of patients with ES. Besides, correlations between clinical characteristics and COL1A2, COL3A1, and TGFBI expression in ES were researched. The univariate analysis revealed that the metastasis stage was related to overall survival. We also found that the expression level of COL3A1 was not associated with metastasis stage, tumor region, age stage, gender stage, and race stage (*p* > 0.01). Besides, correlations between clinical characteristics and COL3A1 expression in ES were researched. The univariate analysis revealed that metastasis stage and COL3A1 expression level are related to overall survival. However, tumor region, age stage, gender stage, and primary site progression were not correlated with overall survival (Table 3). Multivariate analysis also showed that COL3A1 expression level and metastasis stage were an independent risk factor for overall survival in ES.

COL3A1 (collagen type III alpha 1) is an important extracellular matrix protein that was found in 1971 [38]. Type III collagen has many important physiological functions. It is revealed that abnormal overexpression of collagen type III alpha 1 happens in some different types of tumors [39–42]. For example, collagen type III alpha 1 overexpression is associated with poor survival and may be a potential biomarker for early diagnosis of ovarian carcinoma [39]. Engqvist et al. indicated that collagen type III alpha 1 was overexpressed in brain tumors at different stages [40]. Besides, the expression level of COL3A1 can predict the efficacy of neoadjuvant therapy in rectal carcinoma [41]. A previous study also reported that it was differently expressed in a variety of tumors, and its expression is related to tumor immune microenvironment and pan-cancer prognosis. Moreover, it was expected to be further studied as a marker in malignant tumor prognosis and associated tumor immunotherapy [43]. In breast cancer, it was found that methyltransferase-like 3 could target COL3A1 in triple-negative breast cancer cell lines. Methyltransferase-like 3 could suppress the expression of COL3A1 by upregulating its m6A methylation, ultimately inhibiting the metastasis of triple-negative breast cancer cells [44]. In osteosarcoma, it was found that the microRNA-29 family may play a tumor inhibitory role in controlling methotrexate resistance and apoptosis by targeting COL3A1 or MCL1 apoptosis regulators. The development of drugs targeting the microRNA-29 family may provide a new treatment method to overcome the cytotoxicity and drug resistance of osteosarcoma induced by high-dose methotrexate [45].

In recent years, the role of microRNA and its target genes in tumorigenesis and development has been widely studied. Many reports have shown that changes in microRNA and mRNA expression have been found in initial and developing cancers. It is very important to clarify the role of microRNA and mRNA in various human cancers, because the regulation of gene expression may be a new choice for cancer treatment. Our research demonstrated that microRNA-29a and its target gene COL3A1 were involved in the development of ES by several signaling pathways and had prognostic worth. Therefore, overexpression of microRNA-29a or suppression of COL3A1 may have potential therapeutic values in ES patients with metastasis.

## 5. Conclusion

Our study concluded certain mechanisms for the development of ES. Plenty of differentially expressed mRNAs and miRNAs were identified between ES cells and osteoblasts cells. Also, microRNA-29a and its target gene COL3A1 were identified as potential markers of ES. However, these conclusions need further experiments to prove.

## Figures and Tables

**Table 1:** Relationship between the expression level of TGFB1 and clinical characteristics in ES.

Characteristic	Low expression of TGFBI	High expression of TGFBI	*p*
*n*	50	51	
Metastasis, *n* (%)			0.686
No	39 (38.6%)	37 (36.6%)	
Yes	11 (10.9%)	14 (13.9%)	
Tumor region, *n* (%)			1.000
Distal	18 (28.1%)	18 (28.1%)	
Other	2 (3.1%)	1 (1.6%)	
Proximal	12 (18.8%)	13 (20.3%)	
Proximal and distal	0 (0%)	0 (0%)	
Age, *n* (%)			1.000
<18	39 (38.6%)	39 (38.6%)	
≥18	11 (10.9%)	12 (11.9%)	
Gender, *n* (%)			0.372
Female	23 (22.8%)	18 (17.8%)	
Male	27 (26.7%)	33 (32.7%)	
Race, *n* (%)			
American Indian or Alaska Native	0 (0%)	1 (1.3%)	
Asian	3 (3.9%)	4 (5.3%)	
Black or African American	5 (6.6%)	5 (6.6%)	
Native Hawaiian or other Pacific Islander	0 (0%)	0 (0%)	
White	28 (36.8%)	30 (39.5%)	
Age, median (IQR)	15.27 (12.35, 17.69)	15.06 (12.63, 17.77)	0.989

**Table 2:** Relationship between overall survival and the expression level of TGFB1 researched by univariate and multivariate Cox regression.

Characteristics	Total (*N*)	Univariate analysis		Multivariate analysis	
		Hazard ratio (95% CI)	*p* value	Hazard ratio (95% CI)	*p* value
Metastasis (yes vs. no)	99	3.679 (1.964-6.892)	**<0.001**	3.459 (1.438-8.318)	**0.006**
Tumor region (other and proximal and proximal and distal vs. distal)	63	0.473 (0.198-1.127)	0.091	0.547 (0.226-1.321)	0.180
Age (≥18 vs. <18)	99	0.732 (0.325-1.653)	0.454		
TGFBI (high vs. low)	99	0.566 (0.304-1.055)	0.073	0.552 (0.245-1.246)	0.153
Primary site progression (yes vs. no)	50	1.769 (0.864-3.626)	0.119		
Gender (male vs. female)	99	0.976 (0.520-1.832)	0.940		

**Table 3:** Relationship between the expression level of COL1A2 and clinical characteristics in ES.

Characteristic	Low expression of COL1A2	High expression of COL1A2	*p*
*n*	50	51	
Metastasis, *n* (%)			1.000
No	38 (37.6%)	38 (37.6%)	
Yes	12 (11.9%)	13 (12.9%)	
Tumor region, *n* (%)			0.360
Distal	18 (28.1%)	18 (28.1%)	
Other	3 (4.7%)	0 (0%)	
Proximal	13 (20.3%)	12 (18.8%)	
Proximal and distal	0 (0%)	0 (0%)	
Age, *n* (%)			0.139
<18	35 (34.7%)	43 (42.6%)	
≥18	15 (14.9%)	8 (7.9%)	
Gender, *n* (%)			0.626
Female	22 (21.8%)	19 (18.8%)	
Male	28 (27.7%)	32 (31.7%)	
Race, *n* (%)			
American Indian or Alaska Native	1 (1.3%)	0 (0%)	
Asian	5 (6.6%)	2 (2.6%)	
Black or African American	6 (7.9%)	4 (5.3%)	
Native Hawaiian or other Pacific Islander	0 (0%)	0 (0%)	
White	26 (34.2%)	32 (42.1%)	
Age, median (IQR)	15.52 (12.76, 18.8)	13.87 (11.87, 16.65)	0.111

**Table 4:** Relationship between overall survival and the expression level of COL1A2 researched by univariate and multivariate Cox regression.

Characteristics	Total (*N*)	Univariate analysis		Multivariate analysis	
		Hazard ratio (95% CI)	*p* value	Hazard ratio (95% CI)	*p* value
Metastasis (yes vs. no)	99	3.679 (1.964-6.892)	**<0.001**	2.871 (1.253-6.578)	**0.013**
Tumor region (other and proximal and proximal and distal vs. distal)	63	0.473 (0.198-1.127)	0.091	0.518 (0.216-1.241)	0.140
Age (≥18 vs. <18)	99	0.732 (0.325-1.653)	0.454		
COL1A2 (high vs. low)	99	1.026 (0.554-1.900)	0.935		
Gender (male vs. female)	99	0.976 (0.520-1.832)	0.940		
Primary site progression (yes vs. no)	50	1.769 (0.864-3.626)	0.119		

**Table 5:** Relationship between the expression level of COL3A1 and clinical characteristics in ES.

Characteristic	Low expression of COL3A1	High expression of COL3A1	*p*
*n*	50	51	
Metastasis, *n* (%)			0.686
No	39 (38.6%)	37 (36.6%)	
Yes	11 (10.9%)	14 (13.9%)	
Tumor region, *n* (%)			0.920
Distal	19 (29.7%)	17 (26.6%)	
Other	1 (1.6%)	2 (3.1%)	
Proximal	13 (20.3%)	12 (18.8%)	
Proximal and distal	0 (0%)	0 (0%)	
Age, *n* (%)			0.371
<18	41 (40.6%)	37 (36.6%)	
≥18	9 (8.9%)	14 (13.9%)	
Gender, *n* (%)			0.089
Female	25 (24.8%)	16 (15.8%)	
Male	25 (24.8%)	35 (34.7%)	
Race, *n* (%)			
American Indian or Alaska Native	0 (0%)	1 (1.3%)	
Asian	3 (3.9%)	4 (5.3%)	
Black or African American	3 (3.9%)	7 (9.2%)	
Native Hawaiian or other Pacific Islander	0 (0%)	0 (0%)	
White	31 (40.8%)	27 (35.5%)	
Age, median (IQR)	15.12 (12.62, 17.33)	15.1 (12.12, 18.41)	0.973

**Table 6:** Relationship between overall survival and the expression level of COL3A1 researched by univariate and multivariate Cox regression.

Characteristics	Total (*N*)	Univariate analysis		Multivariate analysis	
		Hazard ratio (95% CI)	*p* value	Hazard ratio (95% CI)	*p* value
Metastasis (yes vs. no)	99	3.679 (1.964-6.892)	**<0.001**	3.322 (1.419-7.774)	**0.006**
Tumor region (other and proximal and proximal and distal vs. distal)	63	0.473 (0.198-1.127)	0.091	0.507 (0.212-1.214)	0.127
Age (≥18 vs. <18)	99	0.732 (0.325-1.653)	0.454		
COL3A1 (high vs. low)	99	0.512 (0.273-0.960)	**0.037**	0.522 (0.234-0.865)	**0.002**
Gender (male vs. female)	99	0.976 (0.520-1.832)	0.940		
Primary site progression (yes vs. no)	50	1.769 (0.864-3.626)	0.119		

## Data Availability

The datasets used and/or analyzed during the current study are available from the corresponding author on reasonable request.
